# Firming up your tomato: a natural promoter variation in a MADS-box gene is causing all-flesh tomatoes

**DOI:** 10.1093/jxb/erab442

**Published:** 2022-01-05

**Authors:** Nina Trubanová, Jiaqi Shi, Susanne Schilling

**Affiliations:** School of Biology and Environmental Science and Earth Institute, University College Dublin, Ireland

**Keywords:** AGAMOUS, D class, fruit ripening, gene promoter, MADS domain, tomato

## Abstract

This article comments on:

**Liu L, Zhang K, Bai JR, Lu J, Lu X, Hu J, Pan C, He S, Yuan J, Zhang Y, Zhang M, Guo Y, Wang X, Huang Z, Du Y, Cheng F, Li J.** 2022. All-flesh fruit in tomato is controlled by reduced expression dosage of *AFF* through a structural variant mutation in the promoter. Journal of Experimental Botany **73**, 123–138.

**MADS-domain transcription factors are central regulators of fruit development. Understanding how they function in crop plants can provide valuable insights for research and agriculture. Employing genetic mapping, Liu *et al.* (2021) uncovered a naturally occurring mutation causing the inhibition of locule formation resulting in all-flesh tomatoes. The causal mutation is located in the *cis*-regulatory region of the *SEEDSTICK*-like tomato MADS-box gene termed *ALL FLESH FRUITS* (*AFF*), underlining the vast potential of *cis*-regulatory elements for traditional plant breeding as well as for biotechnology.**

We are all familiar with the 5-a-day recommendation, the advice to eat five portions of fruits or vegetables a day. While staple crops such as wheat and rice are the main providers of calories, this recommendation reflects how important micronutrients are for a healthy well-balanced diet. In a pursuit to supply the world with not only nutritionally rich but also high-yield and shelf-stable foods, plant biologists and breeders are working on improving the present-day variants of food crops. To enable efficient breeding and biotechnological modifications for plant improvement, it is imperative to establish the genetic basis of new traits. In tomato, one family of genes, governing the expression of countless plant hormones, structural proteins, and metabolites, has been shown again and again to have massive potential for crop improvement: MIKC-type MADS-box genes (Box 1).

**Box 1. MADS about tomato: MADS-domain transcription factors regulate all aspects of tomato plant development**

MIKC-type MADS-domain transcription factors comprise a large family of proteins in all seed plants and are involved in virtually all plant developmental processes in all crops, including tomato (Fig. 1) (reviewed in Schilling *et al.*, 2018).

**Fig. 1.**
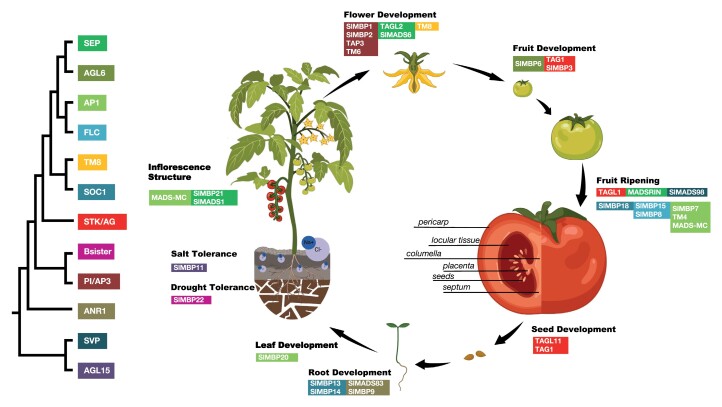
MADS-domain transcription factors in tomato. Tomato life cycle and relevant tomato MADS-domain proteins are depicted on the right. The background of the protein names indicates that they belong to the different subclades. The phylogenetic relationship between different subclades is depicted on the left; the topology of the phylogenetic tree according to Zhao *et al.* (2017); clades are named after Arabidopsis genes except for TM8, which is named after the tomato gene. The figure was created with BioRender.com.

In tomato, MADS-domain transcription factors of all major subclades have been identified (Wang *et al.*, 2019). Proteins of the SUPPRESSOR OF OVEREXPRESSION OF CONSTANS 1 (SOC1) and APETALA1 (AP1) subclade have been shown to be involved in leaf patterning and senescence in tomato (Burko *et al.*, 2013; Xie *et al.*, 2015). Proteins of the SOC1 and ARABIDOPSIS NITRATE REGULATED 1 (ANR1) subclade are involved in tomato root development (Hileman *et al.*, 2006; A. Li *et al.*, 2019, 2020). There is also evidence of MADS-box genes of different subclades involved in salt and drought stress response in tomato (Guo *et al.*, 2016a; Yin *et al.*, 2017; F. Li *et al.*, 2020).

The tomato fruit is the main agricultural product, hence inflorescence, flower development, and fruit ripening controlled by MADS-box genes have been investigated in depth. Floral homeotic *PISTILLATA*/*AP3-* and *AGAMOUS*-like MADS-box genes are key regulators of tomato flower development (de Martino *et al.*, 2006; Guo *et al.*, 2016b; Ocarez and Mejía, 2016; Huang *et al.*, 2017; Cao *et al.*, 2019; Zhang *et al.*, 2019; Liu *et al.*, 2021), which is consistent with their conserved role in other eudicots. *TM8*, which belongs to a subclade not present in the model plant Arabidopsis, is involved in flower development (Daminato *et al.*, 2014). The *SEPALLATA* (*SEP*) subclade of MADS-box genes, which is central to flower induction and floral organ identity in Arabidopsis, has been found to play a central role in tomato inflorescence structure and flower development (Ampomah-Dwamena *et al.*, 2002; Dong *et al.*, 2013; Liu *et al.*, 2014; Soyk *et al.*, 2017). *SEP*-like tomato genes are further central for fruit ripening (Vrebalov *et al.*, 2009; Dong *et al.*, 2013), as are some *FLC-*, *AP1-*, *SHORT VEGETATIVE PHASE-* (*SVP-*), and *SOC1-*like genes (Vrebalov, 2002; Shima *et al.*, 2014; Wang *et al.*, 2014; Xie *et al.*, 2015; Yin *et al.*, 2017, 2018; Zhang *et al.*, 2018; Zhao *et al.*, 2018). Seedlessness in tomato can also be caused by modifications in MADS-box genes (Huang *et al.*, 2017; Zhang *et al.*, 2019; Liu *et al.*, 2021).

## Masterminds over superheroes: locule development in tomato is driven by a MADS-domain transcription factor

The jiggly jelly-like part of a tomato that harbours the seeds, also known as the locule, represents just under a quarter of the fresh weight of tomato (Mounet *et al.*, 2009). Locule development and change of texture go hand in hand with the degradation of the cell wall matrix along with significant changes in gene expression and metabolites governed by a multitude of plant transcription families (reviewed in Quinet *et al.*, 2019). In the present issue, Liu *et al.* (2021) describe a natural tomato mutant that contains no jelly-like locule gel, only locular tissue resembling the central placenta. These all-flesh tomatoes have several advantages for specific applications, as they increase yield from fruit solid content, have a potentially longer shelf life, and might be favoured by some end consumers due to sensory preferences. After performing a thorough morphological analysis, the authors employed several crosses and backcrosses combined with bulked segregation whole-genome sequencing to identify the genetic locus causing the all-flesh phenotype, which they termed *ALL FLESH FRUIT* (*AFF*). Excitingly, the causative mutation is a structural variation (SV), a 416 bp deletion, in the promoter of the tomato MADS-box gene *SlMBP3* (Zhang *et al.*, 2019). Liu *et al.* (2021) demonstrate extensively that it is a decrease in expression of *AFF*/*SlMBP3* that is causative of the mutant phenotype by combining expression analysis, reverse genetics employing CRISPR (clustered regularly interspaced short palindromic repeats), and promoter *in silico* analysis.

*AFF*/*SlMBP3* is an orthologue of the Arabidopsis gene *SEEDSTICK*, a paralogue of the floral homeotic gene *AGAMOUS* (*AG*), which is involved in fruit and seed development in the model plant. Like in Arabidopsis, there are four closely related paralogues in tomato, which belong to the AG/STK lineage. Interestingly, although the four paralogues *AFF*/*SlMBP3*, *TOMATO AGAMOUS-LIKE 1* (*TAGL1*), *TAG1*, and *TAGL11* all have been described to be involved in tomato fruit and seed development (Vrebalov *et al.*, 2009; Gimenez *et al.*, 2016; Ocarez and Mejía, 2016; Huang *et al.*, 2017), none of the paralogues was able to compensate for the decrease in expression of *AFF*/*SlMBP3* in the present study.

Transcription factors have been described as the masterminds amongst domestication genes, in contrast to superheroes, which refer to genes that code for improved enzymes or structural proteins (Martínez-Ainsworth and Tenaillon, 2016). That transcription factors play such a mastermind role in crop domestication is not surprising: changing the sequence or the expression of a single gene coding for a transcription factor has the potential to change the whole plant, inflorescence, or floral architecture, or alter flowering time, fruit-related traits, and more; and countless examples attest to this fact (Martínez-Ainsworth and Tenaillon, 2016; Schilling *et al.*, 2018). The study by Liu *et al.* (2021) is another excellent example of how humans are utilizing changes in MADS-domain transcription factors during domestication and for present-day crop improvement (Schilling *et al.*, 2018). Tomato is a treasure trove for these examples, in all aspects of plant development from inflorescence architecture to fruit ripening and even stress responses (Box 1).

Importantly, the seeds of the *AFF* mutant are viable and germinate, which is in contrast to the CRISPR-generated and previously reported RNAi knockdowns of *AFF*/*SlMBP3* (Zhang *et al.*, 2019). The fact that this now well-characterized mutant is not derived from genetic editing and has an easy-to-use associated genetic marker means that it can be utilized for tomato breeding, even for markets such as the European Union with very strict regulations on genetically modified organisms.

## Promoter editing and *cis*-engineering: configuring the mastermind

More often than not, changes in the coding sequence of a gene can be hugely detrimental, rendering the gene non-functional in all stages of development and plant organs. Therefore, changes in promoters and *cis*-regulatory elements associated with a gene, that alter expression rather than protein sequence, have often been selected for during domestication and might hold a key for future crop improvement (Q. Li *et al.*, 2020). As demonstrated by Liu *et al.* (2021), promoter modifications hold the potential to simultaneously change an economically important plant trait drastically, in this case tissue identity, while still sustaining other vital functions of the gene that were not kept up when the gene was knocked out/down—in this case seed development.

Studying transcription factors such as MADS-domain proteins in model plants and crops over the last three decades brings us the unique advantage that we developed an in-depth understanding of their, often highly conserved, functions. With the simultaneous advancement of gene editing and biotechnological transformation tools for a multitude of different crops as well as species-specific variation databases, we might be able to fine-tune gene expression of promising candidate genes such as those coding for mastermind transcription factors. By engineering their *cis*-regulatory elements, we will be able to achieve quantitative as well as qualitative gene dosage effects that might be impossible to create otherwise. This knowledge can direct us towards building an agriculture fit to serve humanity for the challenges that are waiting for us in the middle and end of the 21st century.
